# Acidic pH Restricts Non‐Tuberculous Mycobacteria Replication

**DOI:** 10.1111/mmi.70060

**Published:** 2026-03-08

**Authors:** Parise K. Lockwood, Luiz Pedro Sorio de Carvalho, Rachel P. J. Lai, Maximiliano G. Gutierrez

**Affiliations:** ^1^ Host‐Pathogen Interactions in Tuberculosis Laboratory The Francis Crick Institute London UK; ^2^ Department of Chemistry The Herbert Wertheim University of Florida Scripps Institute for Biomedical Innovation and Technology Jupiter Florida USA; ^3^ Department of Infectious Diseases Imperial College London London UK

## Abstract

Non‐tuberculous mycobacteria (NTM) are becoming increasingly prevalent in clinical settings with certain environmental species becoming opportunistic in both immunocompetent and immunodeficient hosts. However, the factors that drive this environmental‐to‐opportunistic switch remain poorly understood. NTM are environmental organisms that commonly thrive in acidic niches. Conversely, in host cells, acidic pH acts as a defence mechanism. Here, we investigated how acidic pH impacts NTM replication both in vitro and *in cellulo*. A set of 12 NTM, representing both environmental and opportunistic bacteria, was assessed for growth across a pH range of 4.5–8.0 in vitro, revealing that acid tolerance was species specific. Fluorescent NTM reporters were generated and used to track real‐time live cell intracellular replication within human macrophages where acid tolerant species in vitro replicated with a higher efficiency in cellulo than acid sensitive NTM. Further neutralisation of the phagosomal pH using bafilomycin A1 (BafA1) led to enhanced intracellular replication and survival of NTM. This study demonstrated that specific environmental adaptations may determine the opportunistic potential of NTM whilst acidic pH within the host serves as a key host defence mechanism to restrict NTM growth.

## Graphical abstract

Different nontuberculous mycobacteria (NTM) species display unique replication profiles in acidic pH in vitro which correlates to their replication within human macrophages. Inhibition of the acidic environments within the macrophage enhances bacterial viability emphasising the role of acidic pH during NTM infections.

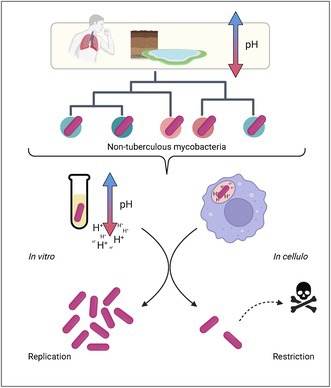

## 1 Introduction

Non‐tuberculous mycobacteria (NTM) comprise all mycobacterial species excluding *Mycobacterium leprae* and those within the *Mycobacterium tuberculosis* (Mtb) complex (Kumar et al. 2024). NTM are primarily saprophytic bacteria that are ubiquitous in terrestrial and aquatic environments. However, the clinical relevance of NTM has gained increasing recognition due to the opportunistic pathogenicity of a few specific species (To et al. 2020). These opportunistic NTM are capable of causing skin, mucosal, pulmonary and disseminated infections, particularly in immunocompromised individuals and the elderly (Ahmed et al. 2020; Kang et al. 2022). Pulmonary disease accounts for approximately 80%–90% of NTM infections, with increased susceptibility observed in individuals with underlying structural lung conditions such as cystic fibrosis (CF), chronic obstructive pulmonary disease (COPD), or prior tuberculosis (Swenson et al. 2018; Griffith et al. 2007; Chindam et al. 2021). Notably, CF patients are affected by NTM pulmonary infection often caused by species within the *Mycobacterium abscessus* (MAB) complex (Prieto et al. 2023; To et al. 2020). The global incidence of pulmonary NTM infections is rising, particularly in countries with a low incidence of Mtb infections, with newly identified species increasingly implicated in opportunistic disease (Winthrop et al. 2020; Williams et al. 2024). Despite the increasing importance of NTM, the host–pathogen interactions underlying NTM infection remain poorly understood, with the mechanisms driving their opportunistic behaviour largely unknown.

Environmental factors may influence the pathogenicity of NTM. Each species exhibits unique biochemical and physiological adaptations that facilitate survival across diverse ecological niches which vary in oxygen concentration, temperature, nutrient availability and pH. Together with temperature, the acidity of the environment is a key factor in bacterial adaptation to specific niches. These adaptations may influence traits linked to virulence and persistence and therefore contribute towards the opportunistic behaviour of NTM within susceptible hosts. NTM are ubiquitously present in the environment, with infections linked to various sources including contaminated water systems, medical equipment and aerosolised particles (Bhanushali et al. 2023). NTM are commonly isolated from soil and water sources characterised by high moisture, acidity and moderate temperatures, with some species capable of survival in pH as low as pH 1.5 (Santos et al. 2007; van Spanning et al. 2022; Walsh et al. 2019). NTM are often more tolerant towards a broader pH range than other bacterial species due to their ability to produce biofilms which contribute to chlorine resistance (Hall‐Stoodley and Lappin‐Scott 1998; September et al. 2004). As a result, these bacteria are commonly found within water distribution systems, increasing the risk of contact and infection to immunocompromised individuals (Lipner et al. 2023; Le Dantec et al. 2002).

NTM are not just exposed to the acidic environments in nature but also encounter acidic pH within the human host. Alveolar macrophages (AM) and airway epithelial cells are primary host cells during pulmonary NTM infections, with AM having the capability to phagocytose and restrict NTM (Awuh and Flo 2016). After phagocytosis, the mycobacteria‐containing phagosome undergoes acidification from an initial pH of ~7.4 to ~4.5, mostly mediated by the vacuolar‐type H^+^ ATPase (V‐ATPase) activity and fusion with lysosomes (Houben et al. 2012; Gomes et al. 1999; Sturgill‐Koszycki et al. 1994). This phagosomal acidification, combined with the toxic environment of the lysosome, can overwhelm bacterial defences and, for some NTM species, reduce viability (Kugadas et al. 2016; Ghanem et al. 2020; Levitte et al. 2016).

Prior research has demonstrated that Mtb employs multiple strategies to survive the acidic conditions of the phagolysosome, thereby facilitating its replication within host cells (Santucci et al. 2022; Buter et al. 2019; Wong et al. 2011; Song et al. 2011; Botella et al. 2017). However, the mechanisms through which NTM adapt to acidic conditions remain poorly understood. Whilst NTM research has primarily focused on a limited number of clinically significant species, the relationship of NTM isolated from both clinical and environmental sources under acidic conditions within host macrophages remains inadequately characterised. Here, we investigated NTM isolated from different environmental sources, including opportunistic pathogens, with a focus on their response towards acidic conditions both in vitro and in human macrophages. We generated first generation fluorescently labelled NTM to facilitate real time live cell tracking of bacterial replication. Strikingly, we discovered that susceptibility to acidic pH is species specific, which directly influences their survival and replication in cellulo. In human macrophages, the impairment of phagosomal acidification significantly enhanced NTM intracellular growth, suggesting that acidic pH serves as a key host defence mechanism in restricting NTM growth. These results provide novel insights into how NTM adapt to acidic pH and persist within host cells, thereby advancing our understanding of their pathogenicity and development towards effective treatments.

## 2 Results

### 2.1 NTM Display Species Specific Responses Towards Acidic pH In Vitro

**FIGURE 1:**
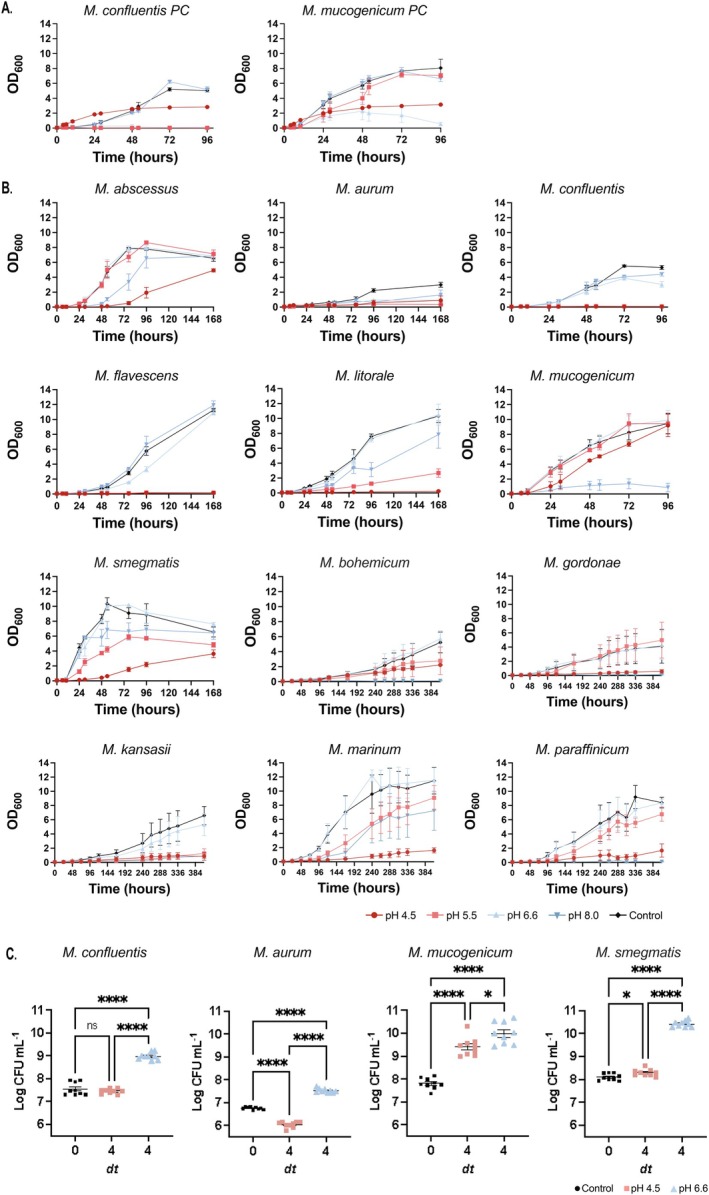
Growth curves of non‐tuberculous mycobacteria in 7H9 media in pH conditions 4.5, 5.5, 6.6 and 8.0. (A) Phosphate citrate buffer and (B) MES (2‐(N‐morpholino)ethanesulphonic acid) (pH 4.5–5.5) and MOPS (3‐(N‐morpholino)propanesulphonic acid) buffer (pH 6.6–8.0) with unbuffered 7H9 media as a control. (C) Number of viable colonies were recorded after 4 doubling times (dt) in pH 4.5 (red) and pH 6.6 (blue) media and compared to the number of viable colonies at the initial inoculum (black). Data is representative *n* = 3 biological replicates with 3 technical replicates ± SEM and visualised on GraphPad Prism v10.1.1. Statistical significance was determined by one‐way ANOVA followed by Dunnett's multiple comparisons test comparing each treatment group to the control group. *p ≤ 0.0332, **p ≤ 0.0021, ***p ≤ 0.0002, ****p ≤ 0.0001 with GraphPad Prism thresholds.

To study if NTM were capable of survival in acidic environments, growth curve experiments were conducted using a subset of NTM species. Twelve NTM species were selected based on their growth dynamics (rapidly or slowly growing mycobacteria (RGM, SGM)), clinical relevance (presence or absence in the clinical setting) and their phylogenetic diversity within the genus to ensure comprehensive representation (Table 1). To investigate the acid tolerance profiles of NTM, in addition to their optimal growth conditions and viability at different pH, we designed an appropriate buffer system to maintain the 7H9 medium at the desired pH to mitigate the risk of the buffer being metabolised by the NTM. Phosphate citrate buffers (Vandal et al. 2008) in addition to 2‐(N‐Morpholino) ethanesulphonic (MES) and 3‐(N‐morpholino) propanesulphonic acid (MOPS) (Piddington et al. 2000; Baker et al. 2014; Gouzy et al. 2021) have previously been used to buffer 7H9 media for mycobacterial growth and therefore were compared to determine the most appropriate system for our experiments (Figure 1). We found that the phosphate citrate buffer changed the growth behaviour of both *Mycobacterium confluentis* and *M. mucogenicum* , two species selected for their different tolerance towards acidic conditions (Figure 1A). In contrast to the MES and MOPs buffered media, the phosphate citrate buffer failed to reproduce the growth dynamics observed in unbuffered 7H9 media at the same pH. Furthermore, the mycobacteria growing in the phosphate citrate buffer resulted in clumping whilst even dispersal was observed within the MES and MOPS buffered medium.

**TABLE 1:** Classification of selected NTM species.

*Mycobacterium* sp	Reference strain	Classification	Source	Clinically relevant
*M. abscessus*	DSMZ 44196	RGM	Unknown source	Opportunistic
*M. aurum*	DSMZ 43999	RGM	Soil	Non‐pathogenic
*M. bohemicum*	DSMZ 44277	SGM	Sputum	Opportunistic
*M. confluentis*	DSMZ 44017	RGM	Sputum	Non‐pathogenic
*M. flavescens*	DSMZ 44017	RGM	Guinea pigs with Mtb	Opportunistic
*M. gordonae*	DSMZ 44160	SGM	Gastric lavage	Opportunistic
*M. kansasii*	DSMZ 44162	SGM	Fatal case	Opportunistic
*M. litorale*	DSMZ 45785	RGM	Soil	Non‐pathogenic
*M. marinum*	DSMZ 44344	SGM	Fish	Pathogenic
*M. mucogenicum*	DSMZ 44124	RGM	Cause of peritonitis	Opportunistic
*M. paraffinicum*	DSMZ 44181	SGM	Soil	Non‐pathogenic
*M. smegmatis*	ATCC 70084	RGM	Derived from mc^2^ 154	Non‐pathogenic

To recapitulate phagosome maturation in vitro, NTM growth curves were measured at pH 4.5, pH 5.5, pH 6.6 and pH 8.0, using 100 mM MES or MOPS buffered medium, whilst unbuffered 7H9 at pH 6.6 served as a positive control. Each mycobacterial species exhibited a distinct response against the different pH conditions (Figure 1B). Average doubling times were calculated via the changes of optical density (OD_600_) over time for each species and condition, excluding conditions in which no significant growth was observed (Table 2). The most optimal pH for mycobacterial growth was confirmed to be pH 6.6. However, *M. mucogenicum* grew almost as efficiently in pH 4.5 (dt = 9.86 h) as pH 6.6 (dt = 9.16 h). Other species were incapable of efficient replication at pH 4.5 such as *Mycobacterium aurum* , *M. confluentis* , *Mycobacterium flavescens* , *Mycobacterium litorale* , *Mycobacterium kansasii* & *Mycobacterium gordonae* . On the other hand, *M. confluentis* & *M. flavescens* grew more efficiently at pH 8.0 with doubling times of 5.85 and 11.88 h respectively in comparison to pH 6.6. RGM displayed a wider pH tolerance in comparison to SGM however growth rate classification was unable to reliably predict how the NTM would survive in acidic pH conditions. Interestingly, the media pH remained unchanged in all mycobacteria tested except *Mycobacterium paraffinicum* in the unbuffered 7H9 control which became increasingly acidic over time (Figure S1B). Altogether, the NTM growth within a pH range of 4.5–8.0 could be categorised into three groups (i) the NTM that could replicate at a similar rate at pH 4.5 as at pH 6.6 (acid tolerant NTM), (ii) the NTM where acidic pH was detrimental for the growth (acid sensitive NTM) and (iii) the NTM that required an initial lag phase to adapt towards the acidic pH environment before replication could occur (acid adapted NTM).

**TABLE 2:** Doubling times of mycobacterial species in 7H9 media buffered at pH 4.5, 5.5, 6.6 and 8.0.

Species	Growth	Doubling time (hours) in pH media					Classification
		4.5	5.5	6.6	8.0	Control 6.6	
*M. abscessus*	RGM	7.75	6.27	4.94	6.87	4.96	Acid‐adapted
*M. aurum*	RGM	NA	NA	13.45	25.91	14.20	Acid‐sensitive
*M. confluentis*	RGM	NA	NA	6.35	5.85	7.24	Acid‐sensitive
*M. flavescens*	RGM	NA	NA	14.47	11.88	11.64	Acid‐sensitive
*M. litorale*	RGM	NA	19.35	12.80	18.72	13.43	Acid‐sensitive
*M. mucogenicum*	RGM	9.86	9.50	9.16	3.81	8.56	Acid‐tolerant
*M. smegmatis*	RGM	13.42	7.53	4.82	2.21	3.71	Acid‐adapted
*M. bohemicum*	SGM	51.45	40.08	55.63	NA	58.15	Acid‐tolerant
*M. gordonae*	SGM	61.65	39.41	51.90	NA	45.54	Acid‐sensitive
*M. kansasii*	SGM	NA	63.19	43.17	49.71	46.09	Acid‐sensitive
*M. marinum*	SGM	49.13	36.89	21.93	23.21	24.64	Acid‐adapted
*M. paraffinicum*	SGM	80.15	33.15	41.92	NA	39.33	Acid‐sensitive

*Note:* The growth curves of each mycobacteria were fitted to the logistic equation Nt = K/1 + (K−N_0_/N_0_)e^−rt^ to determine the average doubling time of each NTM species. Mycobacteria were grown in unbuffered 7H9 media at pH 6.6 as a control. Calculations were performed with the R package growthcurver with *n* = 3 biological replicates. Doubling times marked with NA refer to no significant growth observed.

### 2.2 Acidic pH Reduces Viability for a Subset of NTM

Based on these distinct replication phenotypes observed in acidic conditions, in addition to their suitability for subsequent assays, four representative mycobacterial species were selected: *M. confluentis* , *M. aurum* (acid sensitive) *M. mucogenicum* (acid tolerant) and *M. smegmatis* (acid adapted). We then determined whether the acid sensitive NTM could survive without replicating in an acidic pH environment. To investigate this, we measured viable colony forming units (CFU/mL) between *t* = 0 and after four doubling times (based on the exponential phase at neutral pH previously calculated in Table 2) for all four species cultured in 7H9 media buffered at either pH 4.5 or 6.6 (Figure 1C). The average log_10_ CFU/mL for *M. aurum* at pH 4.5 was 6.03 ± 0.04 (± SEM) showing a significant reduction of viable cells compared to the initial time point (6.76 ± 0.02) suggesting bacterial cell death under the acidic conditions whilst *M. confluentis* was capable of survival in the acidic environment but unable to replicate (pH 4.5 = 7.37 ± 0.03, pH 6.6 = 7.55 ± 0.09). Conversely, *M. mucogenicum* and *M. smegmatis* replicated at pH 4.5 with an average log_10_ CFU/mL of 9.42 ± 0.14 and 8.28 ± 0.05 (± SEM), albeit at a slower rate compared to their growth at pH 6.6 with a log_10_ CFU/mL of 9.99 ± 0.16 and 10.42 ± 0.05 respectively. This emphasised that acidic pH reduced viability and had a bacteriostatic effect towards acid sensitive NTM resulting in reduced survival in vitro. In contrast, acid‐tolerant NTM likely harbour additional mechanisms that enable acid tolerance and growth within acidic pH environments.

### 2.3 NTM Are Phagocytosed by Human Macrophages

Due to the clinical relevance, we wanted to further investigate how acid sensitive and acid tolerant NTM behave within human primary macrophages. While previous studies have shown that certain NTM are internalised by macrophages, these have primarily focused on those clinically relevant, whilst the uptake and internalisation of environmental NTM is less understood (Sturgill‐Koszycki et al. 1994; Crowle et al. 1991; Levitte et al. 2016). To visualise the infection at a cellular level, we generated fluorescently labelled NTM that constitutively express E2Crimson. Human monocyte derived macrophages (HMDMs) were infected with the fluorescently labelled NTM strains at a multiplicity of infection (MOI) of 1 for 2 h to enable phagocytosis before being washed and fixed at 2 and 24 h post infection (hpi). To monitor the localisation of the bacteria within the infection, we additionally labelled the NTM using a surface glycolipid component targeting antibody, lipoarabinomannan (LAM) to allow the visualisation of extracellular mycobacteria within nonpermeabilized HMDMs (Clemens et al. 2002). We observed that the mycobacterial species tested, regardless of growth, clinical relevance or taxonomic positioning, were phagocytosed by human macrophages (Figure S2).

### 2.4 NTM Replication in Acidic Conditions In Vitro Correlates With Intracellular Growth in Macrophages

**FIGURE 2:**
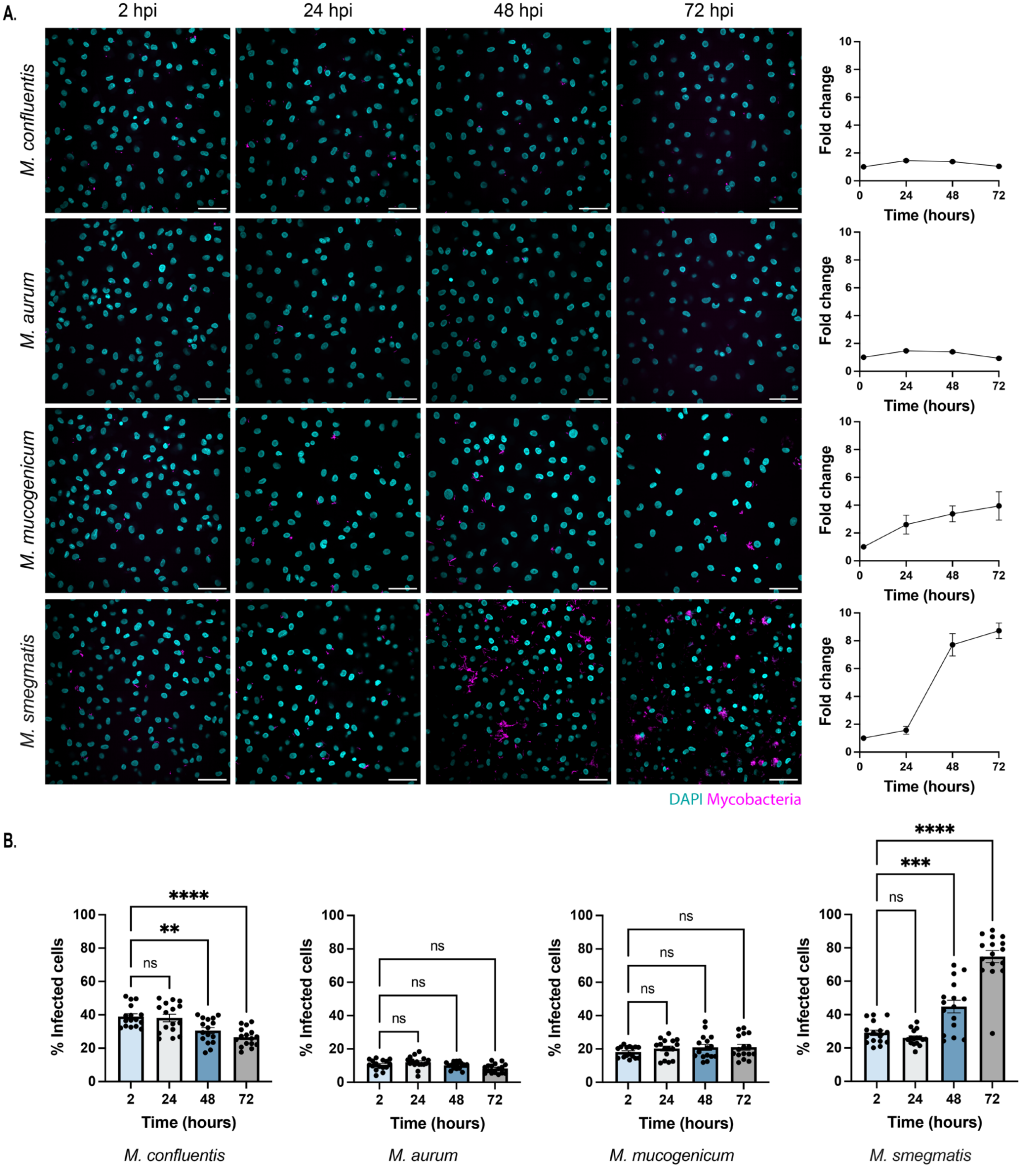
In cellulo replication of mycobacterial species in human monocyte derived macrophages. (A) Representative fluorescent images showing the intracellular replication of *M. confluentis* , *M. aurum* , *M. mucogenicum* and *M. smegmatis* at 2, 24, 48 and 72 hpi. The mean mycobacterial area per host cell was quantified at each time point with the fold change calculated relative to the 2 hpi baseline. (B) The percentage of cells infected with an NTM at each fixed timepoints compared to the total number of cells. Images were captured on the OPERA Phenix microscope with quantification performed on Harmony and GraphPad Prism v 10.1.1. Data represents *n* = 4 ± SEM each with four technical replicates. Statistical significance was determined by one‐way ANOVA followed by Dunnett's multiple comparisons test comparing the mean of each time point to the mean of the 2 hpi baseline. Scale bar represents 50 μm.

We then analysed the replication of the four representative NTM species within HMDMs for 72 h after infection in fixed cells by single cell high content microscopy. Replication was then quantified by comparing the mean mycobacterial area per cell relative to the 2 h baseline (Figures 2A and S3). Consistent with the in vitro observations, the acid sensitive species which were unable to grow in an acidic pH of 4.5, such as *M. aurum* and *M. confluentis*, were unable to replicate intracellularly inside the HMDMs with a fold change of 0.93 ± 0.06 and 1.03 ± 0.04 (SEM) at 72 hpi relative to 2 hpi. In contrast, the species capable of growth in pH 4.5 such as *M. mucogenicum* and *M. smegmatis* had significantly greater intracellular replication in the HMDMs (3.95 ± 1.02 and 8.71 ± 0.56 (± SEM)) during the same period. This growth was particularly notable within the first 48 h after infection before plateauing at 72 hpi. In addition, the growth patterns from the four NTMs correlated with the number of percentage infected cells after infection (Figure 2B). Altogether, these data show that, for the NTM tested, replication in acidic conditions in vitro correlates with replication in cellulo, highlighting that the growth of the NTM in vitro may predict the intracellular growth within human macrophages.

### 2.5 NTM Localise in Acidic Compartments in Human Macrophages

**FIGURE 3:**
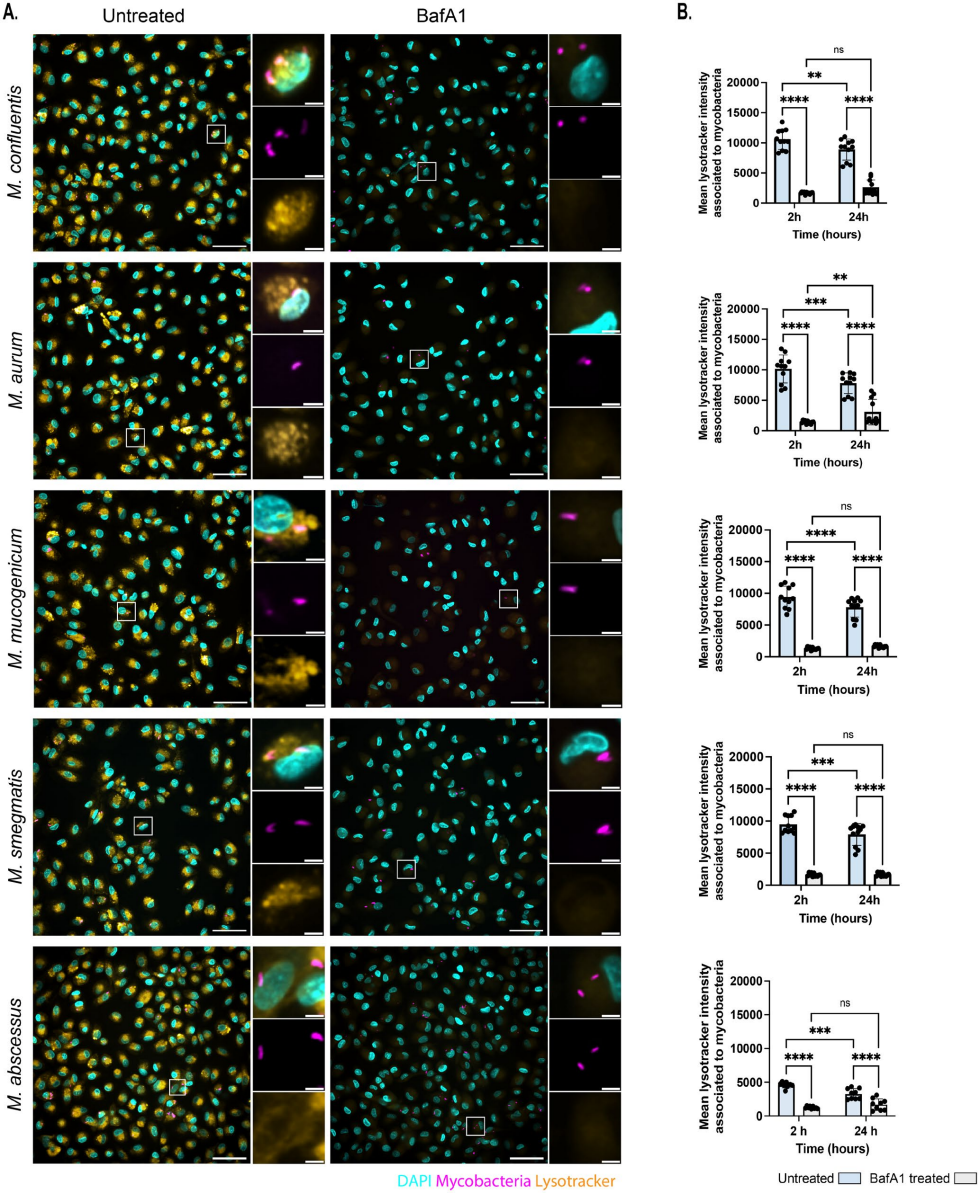
Localisation of mycobacterial species within lysotracker (LT) positive compartments of human macrophages. (A) Representative images showing co‐localisation of LT (Alexa 488, yellow) with *M. confluentis* , *M. aurum* , *M. mucogenicum* , *M. smegmatis* and *M. abcessus* (E2 Crimson, magenta) in untreated and BafA1 treated HMDMs 2hpi. (B) Co‐localisation of mycobacteria with LT was recorded as the mean fluorescent intensity (MFI) at 2 and 24 h post infection. Images were acquired on the OPERA Phenix microscope and analysed on Harmony. Scale bar represents 50 μm and 5 μm in the cropped images. Data representative of three biological replicates ± SD. Statistical significance was determined by two‐way ANOVA followed by the Fisher's LSD test. *p ≤ 0.0332, **p ≤ 0.0021, ***p ≤ 0.0002, ****p ≤ 0.0001 with GraphPad Prism thresholds.

We next sought to define the intracellular localisation of NTM within the macrophages. To determine whether NTM species were trafficked to the acidic compartments in HMDMs, live cell imaging was performed using the lysosomotropic fluorescent probe LysoTracker (LT) that selectively accumulates in acidic intracellular compartments (Santucci et al. 2022). Co‐localisation of LT with the NTM was analysed at 2 and 24 h post infection (Figure 3). We observed that NTM exhibited variable co‐localisation with LT, with mean fluorescent intensity (MFI) values associated with the NTM ranging from 9391 to 10,579 arbitrary units (A.U) at 2 hpi. *M. abscessus* was included for comparison due to its reported ability to disrupt phagosomal maturation and escape into the cytosol of macrophages (Laencina et al. 2018). Consistent with this distinct intracellular behaviour, *M. abscessus* showed a reduced MFI co‐localisation (4586 A.U), providing a contrast to the other NTM species which displayed similar LT association suggesting a similar, comparable intracellular lifestyle. The vacuolar‐type H^+^ ATPase (V‐ATPase) inhibitor bafilomycin A1 (BafA1) neutralises the acidic nature of lysosomes (Bowman et al. 1988). Notably, treatment of NTM‐infected macrophages with BafA1 resulted in a reduction in MFI associated with each NTM by approximately six‐ to seven‐fold, except for *M. abscessus*, which showed a 3.65‐fold reduction at 2 hpi (MFI ranged between 1256 and 16,845 A.U). Altogether, we concluded that in the human macrophage host, NTM were localised in acidic compartments that were sensitive to treatment with BafA1.

### 2.6 Inhibition of Intracellular Acidification Resulted in Enhanced Replication in Human Macrophages

After confirming that NTM localised in acidic compartments and that certain species were unable to replicate within these acidic environments, we next investigated whether intracellular NTM replication was pH dependent. We performed high content live cell imaging to overcome the limitations of static fixed timepoints and gain further insight into the temporal dynamics of NTM replication within human macrophages. Human primary macrophages were inhibited with either 100 nM BafA1 or 15 mM NH_4_Cl, the latter being a weak base that neutralises lysosomal acidity, during the 72 h infection. Macrophage viability was measured using propidium iodide (PI) which detects the loss of plasma membrane integrity indicative of cell death (Jones and Senft 1985; Chen et al. 2025).

**FIGURE 4:**
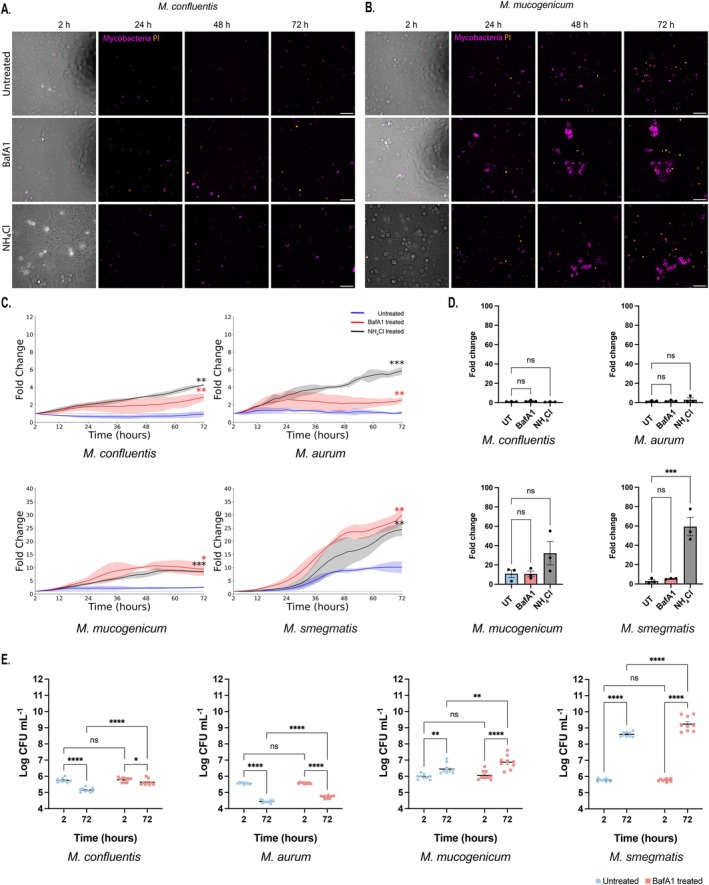
High throughput quantative live imaging of NTM replication in HMDMs. HMDMs were infected with mycobacteria that were treated with either BafA1(red), NH_4_Cl (black) or left untreated (blue). Representative live images of (A) *M. confluentis* and (B) *M. mucogenicum* under the different conditions are shown during the 72 h infection. (C) For each condition, the sum of the mycobacterial area was quantified and compared to the 2 h post infection time point. (D) Cell death of macrophages during the mycobacterial infection was calculated via the fold change between 2 and 72 h post infection in the different treatments based on the number of propidium iodide (PI)‐positive nuclei. (E) Intracellular survival of mycobacterial species within the HMDMs treated with and without BafA1 were measured via the number of viable colonies at 72 h post infection and compared to the number of viable colonies at 2 h post infection. Live replication and cell death data represents *n* = 3 with 12–14 fields analysed per replicate. CFU data represents *n* = 3 ± SEM each with three technical replicates. Image analysis was performed using Harmony software, and data visualisation was conducted using GraphPad Prism v10.1.1, R and Python. Statistical significance live cell replication was assessed using Welch's unpaired t‐test (two‐tailed) comparing treatment groups to the control at 72 hpi. *p ≤ 0.05, **p ≤ 0.01, ***p ≤ 0.001 within Python. For the cell death experiments, statistical significance was determined by one‐way ANOVA followed by Dunnett's multiple comparisons test comparing each treatment group to the control group whilst CFU experiments was determined by two‐way ANOVA followed by the Fisher's LSD test. *p ≤ 0.0332, **p ≤ 0.0021, ***p ≤ 0.0002, ****p ≤ 0.0001 with GraphPad Prism thresholds.

Replication behaviours were consistent with those observed in the fixed timepoint experiments, with the exception that NTM growth continued to increase after 48 h. This extended growth was likely due to the omission of washes and media replacement, thereby minimising disruption of the infection. From the live cell imaging data, all four NTM had a significant increase in replication after treatment with BafA1 and NH_4_Cl, providing further evidence that phagosomal acidification restricts mycobacterial replication within macrophages (Figures 4A–C and S4B and Videos S1–S4). Notably, the acid sensitive NTM had a higher fold‐change of replication in the NH_4_Cl conditions in comparison to BafA1 and untreated conditions ( *M. confluentis* NH_4_Cl fold change 4.25, BafA1 2.84 and untreated 0.94; *M. aurum* NH_4_Cl fold‐change 5.86, BafA1 2.55 and untreated 1.11). In contrast, the acid tolerant NTM had a similar fold‐change between BafA1 and NH_4_Cl compared to untreated ( *M. mucogenicum* NH_4_Cl fold‐change 8.48, BafA1 9.47 and untreated 2.53; *M. smegmatis* NH_4_Cl fold‐change 24.46, BafA1 29.92 and untreated 10.11).

We then analysed by single cell imaging if NTM replication affected macrophage viability using PI where NH_4_Cl and BafA1 treatment alone did not affect macrophage viability (Figure S5A). Interestingly, NTM replication remained largely intracellular and did not appear to have an adverse effect on host cell survival with HMDMs able to sustain large volumes of bacterial burden (Figures 4D and S5B). During the 72 h infection, minimal cell death was observed, except for *M. mucogenicum* and *M. smegmatis* in the NH_4_Cl treated HMDMs ( *M. mucogenicum* fold‐change NH_4_Cl 32.12, *M. smegmatis* fold‐change NH_4_Cl 59.33). Following this host cell death, extracellular bacteria were occasionally observed in the later timepoints; therefore, CFU enumeration was implemented to complement the live cell replication data.

A CFU analysis demonstrated that BafA1 treatment resulted in a greater number of viable colonies after 72 h of infection when compared with untreated HMDMs, supporting the previous observations showing the inhibitory effect of acidic pH on intracellular NTM replication and survival (Figure 4E). However, as both live and dead NTM can retain fluorescence, live cell imaging captures all bacteria and therefore shows replication of acid‐sensitive NTM when the acidic environment is neutralised by BafA1 or NH_4_Cl. In contrast, CFU counts indicate fewer viable bacteria present at 72 h compared to 2 h after infection in the same conditions, suggesting that although some replication was observed, additional phagolysosomal factors, such as hypoxia, reactive oxygen species (ROS) and hydrolases, are also involved (Bussi and Gutierrez 2019). Altogether, physiologic acidification plays an inhibitory role against NTM intracellular replication and viability in a species‐specific manner, with acid‐tolerant species replicating and surviving more efficiently in cellulo than acid‐sensitive species.

## 3 Discussion

It is currently unknown why certain NTM act as opportunistic pathogens while others rarely cause disease. This study explored the relationship between environmental pH and the growth phenotypes of both non‐pathogenic and opportunistic NTM. in vitro growth curve experiments defined distinct, species specific responses to pH across a range of 4.5–8.0, with some displaying pH independent growth, others having impaired growth in acidic pH whilst the remaining required an adaptation period prior to replication. While we could not identify a correlation between the pathogenicity of the species and their ability to tolerate acidic pH, RGM displayed growth in a greater pH range compared to SGM. Further CFU enumeration confirmed that acidic pH in vitro can reduce viability and display bacteriostatic effects to individual NTM, emphasising the heterogeneity in NTM responses.

To provide a more physiologically relevant context, we used *in cellulo* models with human primary macrophages and fluorescent reporters of selected NTM to measure the replication of each mycobacterial species over 72 h. Both fixed timepoints and the use of high‐throughput live cell imaging showed that species capable of replication in an acidic environment in vitro were able to replicate at a higher rate in cellulo in comparison to those that were acid sensitive. After confirmation that NTM were present within lysotracker‐positive compartments, we subsequently inhibited acidification within macrophages with the use of the V‐ATPase inhibitor (BafA1) and a weak base (NH_4_Cl) to understand if acidic pH restricted replication in cellulo. Neutralisation of the intracellular pH resulted in increased viability of all NTM tested, indicating that the restriction of intracellular replication and survival is not exclusively V‐ATPase dependent and reflects broader phagolysosomal acidification. In addition, CFU enumeration showed increased bacterial viability following BafA1 treatment further confirming the importance of phagosomal acidification as a host defence mechanism to restrict NTM growth and survival.

Whilst acidic pH is a major determinant of intracellular replication, NTM survival within macrophages is influenced by other factors including phagosomal acidification, enzymatic activity and host antimicrobial stresses such as ROS, hydrolases and cathepsins (Sturgill‐Koszycki et al. 1994). As these phagolysosomal components were not independently explored within this study, their contribution to intracellular NTM restriction requires further characterisation.

Prior research has identified that certain NTM have developed mechanisms to evade phagosomal acidification. *M. avium* , one of the most common NTM isolated from patients, was observed within non‐acidic compartments due to inhibition of phagosomal acidification via the exclusion of the V‐ATPase (Crowle et al. 1991; Sturgill‐Koszycki et al. 1994; Kelley and Schorey 2003). Similarly, *M. abscessus* has been shown to utilise the ESX‐4 secretion system to rupture the phagosome to localise in the cytosol of macrophages (Laencina et al. 2018; Daher et al. 2025). Early phagosome arrest has also been reported with clinical strains of *M. avium* and *M. fortuitum* enabling persistence and replication within THP‐1 macrophages (Sousa et al. 2019). Notably, *M. kansasii* which was unable to replicate at pH 4.5 in vitro has been shown to replicate in cellulo and in vivo suggesting that acid sensitivity and intracellular survival may act independently depending on species specific evasion strategies (Ghanem et al. 2020; Mussi et al. 2021). Collectively, these studies highlight the diversity of intracellular survival mechanisms utilised by NTM species and the multifactorial restrictive nature of the host environment.

To our knowledge, this is one of the first studies using a range of fluorescent NTM reporters to observe the intracellular behaviour of both environmental and opportunistic species in human primary macrophages. Our findings demonstrate that NTM responses towards environmental factors, such as acidic pH is species‐specific, highlighting the need to have tailored diagnostics and treatment regimens. Given the rising prevalence of NTM infections combined with their intrinsic resistance towards several front line anti‐tuberculosis antibiotics, it is necessary to recognise their heterogeneity to further understand their adaptability towards the host (Dartois and Dick 2024; Subtil et al. 2024). This distinction is crucial as pre‐assumptions and misdiagnosis of NTM infections for tuberculosis can lead to delayed and ineffective treatment due to differences in antibiotic susceptibility. In conclusion, our results highlight the inhibitory role of acidic pH towards NTM replication and survival within human macrophages whilst offering tools that can be further utilised within the NTM field to understand NTM adaptations to the host. Understanding the precise factors contributing to NTM pathogenicity remains complex, requiring further research towards host vulnerabilities, bacterial virulence mechanisms and environmental adaptation processes implicated during infection.

## 4 Material and Methods

### 4.1 Mycobacterial Strains and Culture Conditions

Mycobacterial strains were acquired from the German collection of Microorganisms and Cell Cultures GmbH—DSMZ (Braunschweig, Germany). Mycobacteria were cultured in Middlebrook 7H9 (271310) supplemented with 10% Albumin‐Dextrose‐Catalase (ADC, Mo553‐1VL), 0.2% glycerol and 0.05% tyloxapol. The pH of the media was adjusted with HCl or NaOH where appropriate and buffered using either 100 mM MES (2‐(N‐Morpholino) ethanesulphonic acid) or 100 mM MOPS (3‐(N‐morpholino) propanesulphonic acid). Solid colonies were cultured on Middlebrook 7H10 (262710) containing 10% Oleic Acid Albumin Dextrose Catalase (OADC) and 0.5% glycerol. Fluorescent strains of each mycobacterial species were cultured with the selection marker of 50 μg/mL of hygromycin (Invitrogen, 10687010).

### 4.2 Transformation of NTM

Competent cells were generated as described in (Campo‐Pérez et al. 2021). Briefly, solid isolated colonies were vortexed with 3 mm glass beads before being left to incubate for 20 min in 7H9 media. The bacteria were then transferred to 50 mL of 7H9 and incubated at 37°C for 72 h. The bacteria were heat shocked at 42°C for 2 h before being placed on ice for an hour. The bacteria were pelleted via centrifugation (4000× *g* for 10 min) at 4°C and washed three times in 10% glycerol; the pellet was then vortexed with glass beads and stored in 10% glycerol. The mycobacterial species were electroporated in 0.2 cm Gene‐Pulser cuvettes (Bio‐Rad 165‐2086) with the Bio‐Rad Gene Pulser XCell electroporator to express E2‐Crimson from pTEC19 (Addgene #30178) and grown on solid 7H10 agar plates with a hygromycin selection marker (50 μg/mL).

### 4.3 Growth Measurements of NTM

Mycobacteria were grown to an exponential phase (OD = 0.60–1.00) and seeded at OD = 0.05 in 125 mL Erlenmeyer flasks containing buffered 7H9 media adjusted to pH 4.5, 5.5, 6.6 and 8.0 with unbuffered 7H9 media (pH 6.6) as a positive control. All species were shaken at 100 rpm at 37°C except for *M. abscessus* and *M. marinum* which were grown at 30°C. OD_600_ was regularly measured with an aliquot removed and sterilised to measure extracellular pH. The average doubling times were calculated the logistic growth equation N(t) = K/(1 + ((K−N0)/N0) × (exp(−*r* × *t*))) within the R package growthcurver. To determine the survival of some mycobacterial species in pH 4.5 and 6.6, serial dilutions of cultures were made at the initial timepoint and after 4 doubling times based on the growth of the NTM at pH 6.6 before plated onto solid 7H10 agar plates supplemented with 10% Oleic Acid Albumin Dextrose Catalase (OADC) and 0.5% glycerol and incubated at 37°C.

### 4.4 Primary Human Monocyte Derived Macrophages Culture

Human monocyte‐derived macrophages (HMDMs) were obtained from leukocytes cones provided by the UK National Health Service (NHS). Briefly, the blood was centrifuged with Ficoll‐Paque (GE Healthcare, 17‐5442‐02) at 300× *g* for 60 min to extract the mononuclear cells. These were then washed twice in MACS rinsing solution (Miltenyi, 130‐091‐222) before resuspending in 10 mL of Red Cell Lysis Buffer (Sigma, R7757) for 20 min at 4°C. Cells were washed twice in autoMACs and resuspended in 80 μL of autoMACs with 0.5% bovine serum albumin (MACS/BSA) and 20 μL of CD14 microbeads (Miltenyi, 130‐050‐201) per 10^8^ cells and re‐incubated at 4°C for 15 min. Cells were washed in MACS/BSA and resuspended in 500 μL of MACS/BSA per 10^8^ cells before passing through a pre‐equilibrated LS column (Miltenyi, 130‐042‐401) attached to a QuadroMACs separator magnet (Miltenyi, 130‐090‐976). The columns were washed three times in MACS/BSA before the CD14 positive cells were eluted, centrifuged and resuspended in RPMI 1640 with GlutaMAX and HEPES (Gibco, 72400‐02), 10% foetal bovine serum (Sigma, F7524), and 100 ng/mL of human granulocyte‐macrophage colony‐stimulating factor (hGM‐CSF). Cells were plated in untreated petri dishes at a concentration of 10^6^ cells/mL at 37°C with 5% CO_2_. After three days, fresh media containing GM‐CSF was replenished before the cells were detached from the petri dishes with ice cold MACS rinsing solution on Day 6 and plated for subsequent infections.

### 4.5 Infection With NTM

Mycobacterial cultures (10 mL) were grown to a mid‐exponential phase (OD_600_ = 0.8 ± 0.2) before being centrifuged at 4000 rpm for 5 min and washed twice in PBS before the pellet was shaken vigorously with autoclaved 3 mm glass beads to disperse clumps. The bacterial cultures were resuspended with RPMI 1640 media and centrifuged at 1200× *g* for 5 min to obtain a single cell suspension. The bacterial concentration was determined via OD_600_ measurements with the assumption that an OD = 1 is approximately 1 × 10^8^ bacteria/mL. HMDMs were seeded with a density of 55,000 cells per well with 96 well plates and 150,000 cells per well on a 24 well plate. The HMDMs were then infected with each NTM at an MOI of 1 for 2 h at 37°C and 5% CO_2_ for all experiments. The cells were then washed with PBS to remove the remaining extracellular bacteria, and fresh RPMI 1640 media was added to the cells containing either 100 nM BafA1 (Sigma‐Aldrich B1793) or 15 mM of NH_4_Cl (Sigma‐Aldrich A9434) where stated. For CFU determination, cells were lysed with H_2_O before the lysate was collected at the stated timepoints, serially diluted and plated on 7H10 agar plates supplemented with 10% OADC, 0.5% glycerol and 50 μg/mL of hygromycin.

### 4.6 Indirect Immunofluorescence

Cells were fixed with 4% paraformaldehyde solution (PFA) (Electron Microscopy Sciences, M15710) and quenched in 50 mM NH_4_Cl in PBS for 10 min before permeabilised when required with 0.05% saponin, 0.1% BSA/PBS for an additional 10 min. Antibodies were diluted with 0.1% BSA/PBS to stain the cells with mycobacteria anti‐LAM Monoclonal Antibody (Invitrogen, MA5‐33311, 1:50), Alexa Fluor 488 Phalloidin (CST, 8878S, 1:20) and Alexa‐Fluor‐488 anti‐mouse/human‐Mac‐2 (Galectin‐3) antibody (BioLegend, 125410, 1:200) and incubated for 1 h. Washes with PBS were applied in between primary and secondary antibodies and then stained with DAPI (ThermoFisher, D1306 1:10000) for 10 min. Samples were then washed and mounted.

### 4.7 High Content Imaging and Analysis

Imaging was performed using the Opera Phenix Microscope (PerkinElmer) equipped with a 40× 1.1 NA water‐immersion objective. Images for live cell replication were acquired across 12–14 fields per well, with a 10% overlap between adjacent fields, approximately every 2.5 h over a 72 h period. To assess live cell intracellular replication of NTM and host cell death, the growth media was replaced with RPMI 1640 containing 0.4 μg/mL propidium iodide (PI; Abcam, ab14083), 100 nM BafA1 (Sigma‐Aldrich B1793) or 15 mM of NH_4_Cl (Sigma‐Aldrich A9434) where stated. For co‐localisation of the bacteria with lysotracker, HMDMs were infected as mentioned and washed in PBS before adding fresh RPMI 1640 containing 200 nM LysoTracker Green DND‐26 (LTR; Invitrogen, L7526) and NucBlue ReadyProbes Reagent (Invitrogen, R37605) to stain the cells 40 min prior imaging.

Segmentation and analysis were performed using Harmony software (Perkin Elmer, version 4.9) using maximum projection of the images across the *z*‐planes that were approximately 1 μm apart. For live cell imaging, the cellular area was segmented via the ‘Find Texture Regions’ which was trained within the brightfield channel. The sum of PI positive nuclei at each timeframe was calculated via the ‘Find Nuclei’ in the PI channel before the fold change of the number of PI nuclei for each timeframe was compared to the 2 hpi baseline to calculate cell viability. For the lysotracker and fixed replication experiments, single cell segmentation was performed with the ‘Find Nuclei’ and ‘Find Cytoplasm’ function in the DAPI, Galectin 3‐Alexa 488 or LysoTracker Green DND‐26 channel. Single cell bacterial segmentation was achieved using the ‘Find Spots’ function. Intracellular replication was calculated by measuring the mean spot area per infected cell/cellular area at each time point before calculating the fold change of the mean NTM area per infected cell/cellular area relative to the 2 hpi baseline. Segmentation pipelines for fixed and live replication are visually shown in Figures S3 and S4A.

### 4.8 Confocal Imaging

The Leica SP8 inverted confocal microscope with a 63× 1.4 NA oil immersion objective was used to capture the association of the mycobacterial LAM antibody (Invitrogen, MA5‐33311) with mycobacterial species. Acquired images were a maximum z projection ranging from 4.18 to 9.25 μm. Images were analysed in Fiji v1.54f.

### 4.9 Bioinformatics and Statistics

Graphs and statistical analysis were performed with GraphPad Prism (Version 10.1.1), R (Version 4.3.2) or Python.

## Author Contributions

Conceptualization: M.G.G., L.P.S.C.; Methodology: P.K.L.; Investigation: P.K.L., M.G.G.; Visualization: P.K.L., M.G.G.; Supervision: M.G.G., R.P.J.L.; writing original draft: P.K.L., M.G.G.; Writing review and editing: all.

## Funding

This work was supported by the Francis Crick Institute (to M.G.G.), which receives its core funding from Cancer Research UK (CC2081), the UK Medical Research Council (CC2081), the Wellcome Trust (CC2081) and the UKRI‐BBSRC (BB/X000613/1).

## Disclosure

The graphical abstract was created using BioRender.com.

## Ethics Statement

Leukocyte cones (NC24) were obtained from the National Health Service (NHS) Blood and Transplant Service from healthy donors with informed consent. All experimental procedures were conducted at the Francis Crick Institute under a Human Tissue Act licence (Licence No. 12650) and in accordance with relevant ethical and regulatory guidelines.

## Conflicts of Interest

The authors declare no conflicts of interest.

## Supporting information


**Figure S1:** (A) External pH measurements of *M. confluentis* and *M. mucogenicum* in 7H9 media in pH conditions 4.5, 5.5, 6.6 and 8.0 with a phosphate citrate buffer. (B) External pH measurements taken during the growth curves of each mycobacterial species in MES and MOPS buffer with the positive control being unbuffered 7H9 media at pH 6.6. Data is representative *n* = 3 biological replicates ± SEM and visualised on GraphPad Prism v10.1.1.
**Figure S2:** Uptake of mycobacterial species in human monocyte derived macrophages. Representative images showing intracellular fluorescent mycobacterial species (arrows) with extracellular bacteria (arrowheads) detected with an anti‐LAM antibody 2 h post infection. Images were acquired on the Leica SP8 confocal microscope with a maximum z projection ranging from 4.18 to 9.25 μm before analysed in Fiji v1.54f with the orthogonal view of YZ and XZ. Scale bar represents 20 μm.
**Figure S3:** Segmentation workflow of fixed replication imaging experiments. Image acquired is a fixed image of *M. mucogenicum* 48 h post infection with cell boundaries segmented with galectin 3 Alexa 488. Acquired images were max z‐projections of 4 μm. Infected cells were cells defined as those containing intracellular E2Crimson fluorescent ‘spots’. Segmentation was performed on the Harmony software 4.9.
**Figure S4:** (A) Segmentation workflow of the live cell replication experiment. Image acquired is maximum z projection of a live cell image of *M. aurum* in untreated HMDMs. (B) Representative images of the live cell replication of *M. aurum* and *M. smegmatis* within untreated, NH_4_Cl and BafA1 treated HMDMs. Images acquired on the OPERA Phenix and segmented on the Harmony software 4.9. Scale bar represents 50 μM.
**Figure S5:** Cell death of untreated, BafA1 treated and NH_4_Cl treated HMDMs cell over 72 h. (A) Cell death was calculated based on the fold change of the number of propidium iodide (PI)‐positive nuclei between 2 and 72 hpi. The number of PI positive nuclei for each NTM species in each condition at (B) 2 h post infection and (C) 72 h post infection. Data representative of three biological replicates whilst statistical significance was determined by (A) one‐way ANOVA; (B, C) Two‐way ANOVA followed by Dunnett's multiple comparisons test comparing each treatment group to the control group. Image analysis was performed using Harmony software, and data visualisation was conducted using GraphPad Prism v10.1.1. Scale bar represents 50 μm.

**Videos S1–S4:** Live cell imaging of NTM replication ( *M. confluentis (S1)* , *M. aurum (S2)* , *M. mucogenicum (S3)* & *M. smegmatis (S4)* ) in human macrophages treated with BafA1 and NH_4_Cl. Images were obtained approximately every 2.5 h over a 72 h period by the OPERA Phenix microscope with 40× 1.1 NA water‐immersion objective with maximum *z* projection. Scale bar represents 50 μM.

## Data Availability

The data that support the findings of this study are available from the corresponding author upon reasonable request.

## References

[mmi70060-bib-0001] Ahmed, I. , S. Tiberi , J. Farooqi , et al. 2020. “Non‐Tuberculous Mycobacterial Infections—A Neglected and Emerging Problem.” International Journal of Infectious Diseases 92: S46–S50. 10.1016/j.ijid.2020.02.022. 32114200

[mmi70060-bib-0002] Awuh, J. A. , and T. H. Flo . 2016. “Molecular Basis of Mycobacterial Survival in Macrophages.” Cellular and Molecular Life Sciences: CMLS 74, no. 9: 1625–1648. 10.1007/s00018-016-2422-8. 27866220 PMC11107535

[mmi70060-bib-0044] Baker, J. J. , B. K. Johnson , and R. B. Abramovitch . 2014. “Slow Growth of *Mycobacterium tuberculosis* at Acidic pH Is Regulated by phoPR and Host‐Associated Carbon Sources.” Molecular Microbiology 94, no. 1: 56–69. 10.1111/mmi.12688. 24975990 PMC4177513

[mmi70060-bib-0041] Bhanushali, J. , U. Jadhav , B. Ghewade , and P. Wagh . 2023. “Unveiling the Clinical Diversity in Nontuberculous Mycobacteria (NTM) Infections: A Comprehensive Review.” Cureus. 10.7759/cureus.48270. PMC10695653 38054150

[mmi70060-bib-0003] Botella, H. , J. Vaubourgeix , M. H. Lee , et al. 2017. “ *Mycobacterium tuberculosis* Protease MarP Activates a Peptidoglycan Hydrolase During Acid Stress.” EMBO Journal 36, no. 4: 536–548. 10.15252/embj.201695028. 28057704 PMC5437814

[mmi70060-bib-0004] Bowman, E. J. , A. Siebers , and K. Altendorf . 1988. “Bafilomycins: A Class of Inhibitors of Membrane ATPases From Microorganisms, Animal Cells, and Plant Cells.” Proceedings of the National Academy of Sciences of the United States of America 85, no. 21: 7972–7976. 10.1073/pnas.85.21.7972. 2973058 PMC282335

[mmi70060-bib-0005] Bussi, C. , and M. G. Gutierrez . 2019. “ *Mycobacterium tuberculosis* Infection of Host Cells in Space and Time.” FEMS Microbiology Reviews 43, no. 4: 341–361. 10.1093/femsre/fuz006. 30916769 PMC6606852

[mmi70060-bib-0006] Buter, J. , T.‐Y. Cheng , M. Ghanem , et al. 2019. “ *Mycobacterium tuberculosis* Releases an Antacid That Remodels Phagosomes.” Nature Chemical Biology 15, no. 9: 889–899. 10.1038/s41589-019-0336-0. 31427817 PMC6896213

[mmi70060-bib-0048] Campo‐Pérez, V. , M. d. M. Cendra , E. Julián , and E. Torrents . 2021. “Easily Applicable Modifications to Electroporation Conditions Improve the Transformation Efficiency Rates for Rough Morphotypes of Fast‐Growing Mycobacteria.” New Biotechnology 63: 10–18. 10.1016/j.nbt.2021.02.003. 33636348

[mmi70060-bib-0007] Chen, D. , A. Fearns , and M. G. Gutierrez . 2025. “ *Mycobacterium tuberculosis* Phagosome Ca^2+^ Leakage Triggers Multimembrane ATG8/LC3 Lipidation to Restrict Damage in Human Macrophages.” Science Advances 11, no. 13: eadt3311. 10.1126/sciadv.adt3311. 40138395 PMC11939036

[mmi70060-bib-0008] Chindam, A. , S. Vengaldas , V. R. Srigiri , et al. 2021. “Challenges of Diagnosing and Treating Non‐Tuberculous Mycobacterial Pulmonary Disease [NTM‐PD]: A Case Series.” Journal of Clinical Tuberculosis and Other Mycobacterial Diseases 25: 100271. 10.1016/j.jctube.2021.100271. 34541338 PMC8441069

[mmi70060-bib-0009] Clemens, D. L. , B.‐Y. Lee , and M. A. Horwitz . 2002. “The *Mycobacterium tuberculosis* Phagosome in Human Macrophages Is Isolated From the Host Cell Cytoplasm.” Infection and Immunity 70, no. 10: 5800–5807. 10.1128/IAI.70.10.5800-5807.2002. 12228310 PMC128330

[mmi70060-bib-0010] Crowle, A. J. , R. Dahl , E. Ross , and M. H. May . 1991. “Evidence That Vesicles Containing Living, Virulent *Mycobacterium tuberculosis* or *Mycobacterium avium* in Cultured Human Macrophages Are Not Acidic.” Infection and Immunity 59, no. 5: 1823–1831. 1902198 10.1128/iai.59.5.1823-1831.1991 PMC257922

[mmi70060-bib-0011] Daher, W. , V. Le Moigne , Y. Tasrini , et al. 2025. “Deletion of ESX‐3 and ESX‐4 Secretion Systems in *Mycobacterium abscessus* Results in Highly Impaired Pathogenicity.” Communications Biology 8, no. 1: 166. 10.1038/s42003-025-07572-4. 39900631 PMC11791044

[mmi70060-bib-0012] Dartois, V. , and T. Dick . 2024. “Therapeutic Developments for Tuberculosis and Nontuberculous Mycobacterial Lung Disease.” Nature Reviews. Drug Discovery 23, no. 5: 381–403. 10.1038/s41573-024-00897-5. 38418662 PMC11078618

[mmi70060-bib-0013] Ghanem, M. , J.‐Y. Dubé , J. Wang , et al. 2020. “Heterologous Production of 1‐Tuberculosinyladenosine in *Mycobacterium kansasii* Models Pathoevolution Towards the Transcellular Lifestyle of *Mycobacterium tuberculosis* .” MBio 11, no. 5: e02645‐20. 10.1128/mBio.02645-20. 33082253 PMC7587436

[mmi70060-bib-0014] Gomes, M. S. , S. Paul , A. L. Moreira , R. Appelberg , M. Rabinovitch , and G. Kaplan . 1999. “Survival of *Mycobacterium avium* and *Mycobacterium tuberculosis* in Acidified Vacuoles of Murine Macrophages.” Infection and Immunity 67, no. 7: 3199–3206. 10377091 10.1128/iai.67.7.3199-3206.1999 PMC116496

[mmi70060-bib-0045] Gouzy, A. , C. Healy , K. A. Black , K. Y. Rhee , and S. Ehrt . 2021. “Growth of *Mycobacterium tuberculosis* at Acidic pH Depends on Lipid Assimilation and Is Accompanied by Reduced GAPDH Activity.” Proceedings of the National Academy of Sciences 118, no. 32. 10.1073/pnas.2024571118. PMC8364206 34341117

[mmi70060-bib-0015] Griffith, D. E. , T. Aksamit , B. A. Brown‐Elliott , et al. 2007. “An Official ATS/IDSA Statement: Diagnosis, Treatment, and Prevention of Nontuberculous Mycobacterial Diseases.” American Journal of Respiratory and Critical Care Medicine 175, no. 4: 367–416. 10.1164/rccm.200604-571ST. 17277290

[mmi70060-bib-0016] Hall‐Stoodley, L. , and H. Lappin‐Scott . 1998. “Biofilm Formation by the Rapidly Growing Mycobacterial Species *Mycobacterium fortuitum* .” FEMS Microbiology Letters 168, no. 1: 77–84. 10.1111/j.1574-6968.1998.tb13258.x. 9812366

[mmi70060-bib-0017] Houben, D. , C. Demangel , J. van Ingen , et al. 2012. “ESX‐1‐Mediated Translocation to the Cytosol Controls Virulence of Mycobacteria.” Cellular Microbiology 14, no. 8: 1287–1298. 10.1111/j.1462-5822.2012.01799.x. 22524898

[mmi70060-bib-0018] Jones, K. H. , and J. A. Senft . 1985. “An Improved Method to Determine Cell Viability by Simultaneous Staining With Fluorescein Diacetate‐Propidium Iodide.” Journal of Histochemistry and Cytochemistry 33, no. 1: 77–79. 10.1177/33.1.2578146. 2578146

[mmi70060-bib-0019] Kang, M. , H. W. Kim , A.‐R. Yu , et al. 2022. “Comparison of Macrophage Immune Responses and Metabolic Reprogramming in Smooth and Rough Variant Infections of *Mycobacterium mucogenicum* .” International Journal of Molecular Sciences 23, no. 5: 2488. 10.3390/ijms23052488. 35269631 PMC8910348

[mmi70060-bib-0046] Kelley, V. A. , and J. S. Schorey . 2003. “Mycobacterium's Arrest of Phagosome Maturation in Macrophages Requires Rab5 Activity and Accessibility to Iron.” Molecular Biology of the Cell 14, no. 8: 3366–3377. 10.1091/mbc.e02-12-0780. 12925769 PMC181573

[mmi70060-bib-0020] Kugadas, A. , E. A. Lamont , J. P. Bannantine , et al. 2016. “A *Mycobacterium avium* Subsp. Paratuberculosis Predicted Serine Protease Is Associated With Acid Stress and Intraphagosomal Survival.” Frontiers in Cellular and Infection Microbiology 6: 85. 10.3389/fcimb.2016.00085. 27597934 PMC4992679

[mmi70060-bib-0021] Kumar, K. , A. Ponnuswamy , T. G. Capstick , et al. 2024. “Non‐Tuberculous Mycobacterial Pulmonary Disease (NTM‐PD): Epidemiology, Diagnosis and Multidisciplinary Management.” Clinical Medicine 24, no. 1: 100017. 10.1016/j.clinme.2024.100017. 38387207 PMC11024839

[mmi70060-bib-0022] Laencina, L. , V. Dubois , V. Le Moigne , et al. 2018. “Identification of Genes Required for *Mycobacterium abscessus* Growth In Vivo With a Prominent Role of the ESX‐4 Locus.” Proceedings of the National Academy of Sciences of the United States of America 115, no. 5: E1002–E1011. 10.1073/pnas.1713195115. 29343644 PMC5798338

[mmi70060-bib-0023] Le Dantec, C. , J.‐P. Duguet , A. Montiel , N. Dumoutier , S. Dubrou , and V. Vincent . 2002. “Chlorine Disinfection of Atypical Mycobacteria Isolated From a Water Distribution System.” Applied and Environmental Microbiology 68, no. 3: 1025–1032. 10.1128/AEM.68.3.1025-1032.2002. 11872446 PMC123737

[mmi70060-bib-0024] Levitte, S. , K. N. Adams , R. D. Berg , C. L. Cosma , K. B. Urdahl , and L. Ramakrishnan . 2016. “Mycobacterial Acid Tolerance Enables Phagolysosomal Survival and Establishment of Tuberculous Infection In Vivo.” Cell Host & Microbe 20, no. 2: 250–258. 10.1016/j.chom.2016.07.007. 27512905 PMC4985559

[mmi70060-bib-0025] Lipner, E. M. , J. P. French , R. A. Mercaldo , et al. 2023. “The Risk of Pulmonary NTM Infections and Water‐Quality Constituents Among Persons With Cystic Fibrosis in the United States, 2010–2019.” Environmental Epidemiology 7, no. 5: e266. 10.1097/EE9.0000000000000266. 37840858 PMC10569765

[mmi70060-bib-0026] Mussi, V. O. , T. L. B. V. Simão , F. M. Almeida , et al. 2021. “A Murine Model of *Mycobacterium kansasii* Infection Reproducing Necrotic Lung Pathology Reveals Considerable Heterogeneity in Virulence of Clinical Isolates.” Frontiers in Microbiology 12: 718477. 10.3389/fmicb.2021.718477. 34504483 PMC8422904

[mmi70060-bib-0043] Piddington, D. L. , A. Kashkouli , and N. A. Buchmeier . 2000. “Growth of *Mycobacterium tuberculosis* in a Defined Medium Is Very Restricted by Acid pH and Mg2+Levels.” Infection and Immunity 68, no. 8: 4518–4522. 10.1128/iai.68.8.4518-4522.2000. 10899850 PMC98362

[mmi70060-bib-0027] Prieto, M. D. , M. E. Alam , A. N. Franciosi , and B. S. Quon . 2023. “Global Burden of Nontuberculous Mycobacteria in the Cystic Fibrosis Population: A Systematic Review and Meta‐Analysis.” ERJ Open Research 9, no. 1: 00336–02022. 10.1183/23120541.00336-2022. PMC9808535 36605902

[mmi70060-bib-0028] Santos, R. , J. Fernandes , N. Fernandes , F. Oliveira , and M. Cadete . 2007. “ *Mycobacterium parascrofulaceum* in Acidic Hot Springs in Yellowstone National Park.” Applied and Environmental Microbiology 73, no. 15: 5071–5073. 10.1128/AEM.00353-07. 17557859 PMC1951044

[mmi70060-bib-0029] Santucci, P. , B. Aylan , L. Botella , et al. 2022. “Visualizing Pyrazinamide Action by Live Single‐Cell Imaging of Phagosome Acidification and *Mycobacterium tuberculosis* pH Homeostasis.” MBio 13, no. 2: e0011722. 10.1128/mbio.00117-22. 35323041 PMC9040869

[mmi70060-bib-0030] September, S. M. , V. S. Brözel , and S. N. Venter . 2004. “Diversity of Nontuberculoid Mycobacterium Species in Biofilms of Urban and Semiurban Drinking Water Distribution Systems.” Applied and Environmental Microbiology 70, no. 12: 7571–7573. 10.1128/AEM.70.12.7571-7573.2004. 15574964 PMC535200

[mmi70060-bib-0031] Song, H. , J. Huff , K. Janik , et al. 2011. “Expression of the ompATb Operon Accelerates Ammonia Secretion and Adaptation of *Mycobacterium tuberculosis* to Acidic Environments.” Molecular Microbiology 80, no. 4: 900–918. 10.1111/j.1365-2958.2011.07619.x. 21410778 PMC3091969

[mmi70060-bib-0047] Sousa, S. , V. Borges , I. Joao , J. P. Gomes , and L. Jordao . 2019. “Nontuberculous Mycobacteria Persistence in a Cell Model Mimicking Alveolar Macrophages.” Microorganisms 7, no. 5: 113. 10.3390/microorganisms7050113. 31035520 PMC6560506

[mmi70060-bib-0032] Sturgill‐Koszycki, S. , P. H. Schlesinger , P. Chakraborty , et al. 1994. “Lack of Acidification in Mycobacterium Phagosomes Produced by Exclusion of the Vesicular Proton‐ATPase.” Science (New York, N.Y.) 263, no. 5147: 678–681. 10.1126/science.8303277. 8303277

[mmi70060-bib-0033] Subtil, F. , T. Machado , H. Douglas , et al. 2024. “A Macroevolution‐Inspired Approach to Reveal Novel Antibiotic Resistance Mechanisms.” eLife 13: RP101940. 10.7554/eLife.101940.1. PMC13246000 42253228

[mmi70060-bib-0034] Swenson, C. , C. S. Zerbe , and K. Fennelly . 2018. “Host Variability in NTM Disease: Implications for Research Needs.” Frontiers in Microbiology 9: 2901. 10.3389/fmicb.2018.02901. 30559727 PMC6286975

[mmi70060-bib-0035] To, K. , R. Cao , A. Yegiazaryan , J. Owens , and V. Venketaraman . 2020. “General Overview of Nontuberculous Mycobacteria Opportunistic Pathogens: *Mycobacterium avium* and *Mycobacterium abscessus* .” Journal of Clinical Medicine 9, no. 8: 2541. 10.3390/jcm9082541. 32781595 PMC7463534

[mmi70060-bib-0036] van Spanning, R. J. M. , Q. Guan , C. Melkonian , et al. 2022. “Methanotrophy by a Mycobacterium Species That Dominates a Cave Microbial Ecosystem.” Nature Microbiology 7, no. 12: 2089–2100. 10.1038/s41564-022-01252-3. 36329197

[mmi70060-bib-0042] Vandal, O. H. , L. M. Pierini , D. Schnappinger , C. F. Nathan , and S. Ehrt . 2008. “A Membrane Protein Preserves Intrabacterial pH in Intraphagosomal *Mycobacterium tuberculosis* .” Nature Medicine 14, no. 8: 849–854. 10.1038/nm.1795. PMC2538620 18641659

[mmi70060-bib-0037] Walsh, C. M. , M. J. Gebert , M. Delgado‐Baquerizo , F. T. Maestre , and N. Fierer . 2019. “A Global Survey of Mycobacterial Diversity in Soil.” Applied and Environmental Microbiology 85, no. 17: e01180‐19. 10.1128/AEM.01180-19. 31253672 PMC6696970

[mmi70060-bib-0038] Williams, P. M. , R. H. Pratt , W. L. Walker , S. F. Price , R. J. Stewart , and P.‐J. I. Feng . 2024. “Tuberculosis—United States, 2023.” Morbidity and Mortality Weekly Report 73, no. 12: 265–270. 10.15585/mmwr.mm7312a4. 38547024 PMC10986816

[mmi70060-bib-0039] Winthrop, K. L. , T. K. Marras , J. Adjemian , H. Zhang , P. Wang , and Q. Zhang . 2020. “Incidence and Prevalence of Nontuberculous Mycobacterial Lung Disease in a Large U.S. Managed Care Health Plan, 2008‐2015.” Annals of the American Thoracic Society 17, no. 2: 178–185. 10.1513/AnnalsATS.201804-236OC. 31830805 PMC6993793

[mmi70060-bib-0040] Wong, D. , H. Bach , J. Sun , Z. Hmama , and Y. Av‐Gay . 2011. “ *Mycobacterium tuberculosis* Protein Tyrosine Phosphatase (PtpA) Excludes Host Vacuolar‐H+–ATPase to Inhibit Phagosome Acidification.” Proceedings of the National Academy of Sciences of the United States of America 108, no. 48: 19371–19376. 10.1073/pnas.1109201108. 22087003 PMC3228452

