# Supplementary material for: Acidic pH Restricts Non‐Tuberculous Mycobacteria Replication
Source: Mol Microbiol. 2026 Mar 8;125(5):375–88. doi: 10.1111/mmi.70060 (PMC13135906; doi:10.1111/mmi.70060)

Supplementary Figure 1

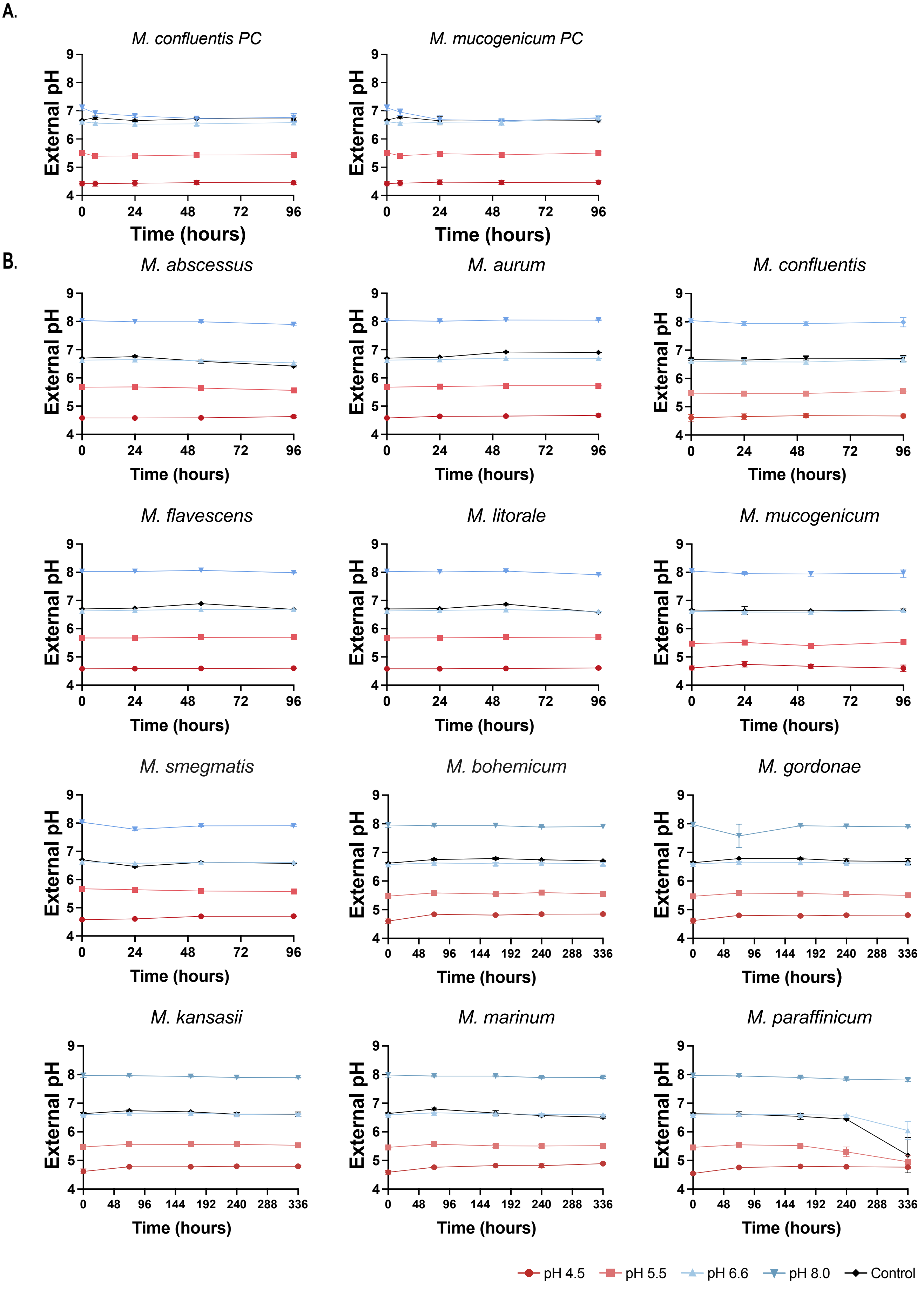

Supplementary Figure 2

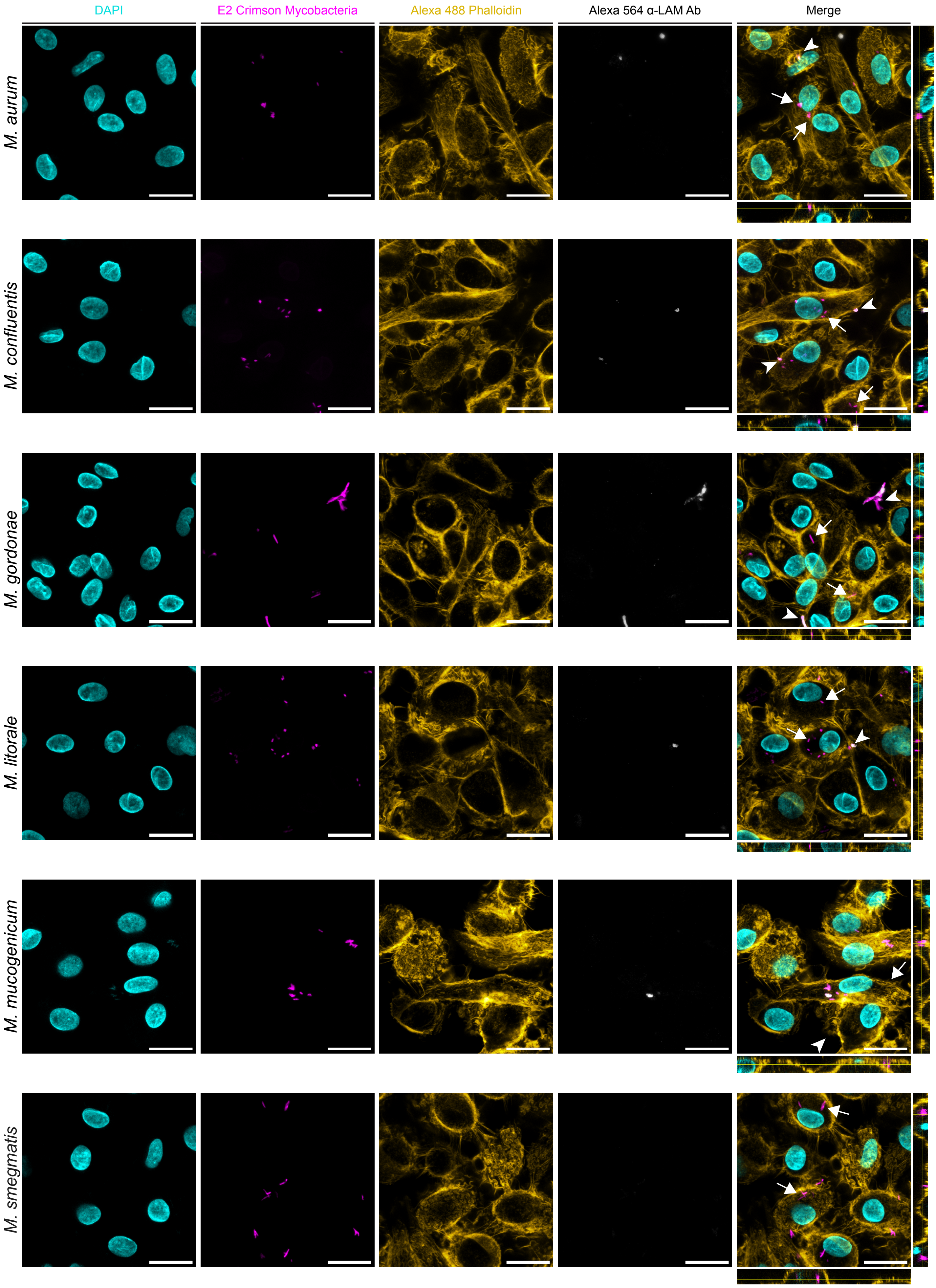

Supplementary Figure 3

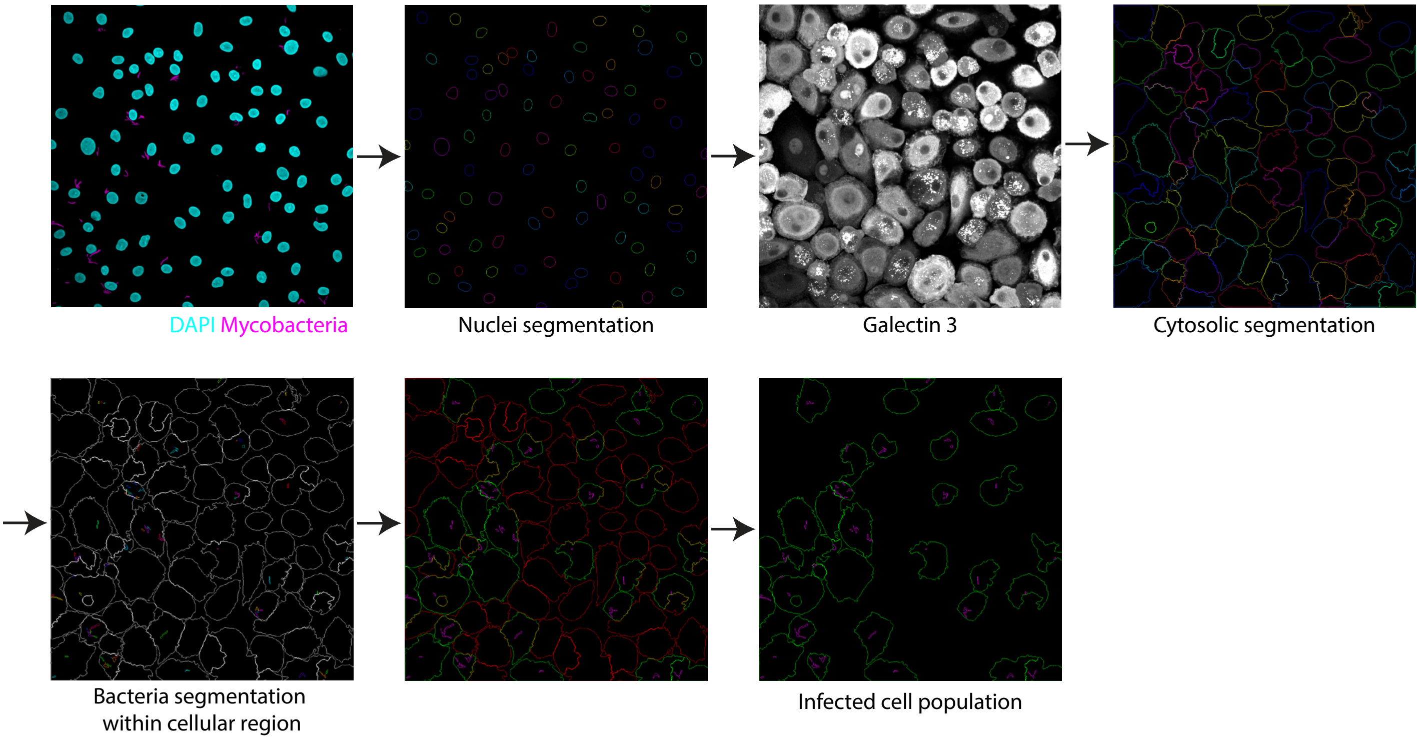

Supplementary Figure 4

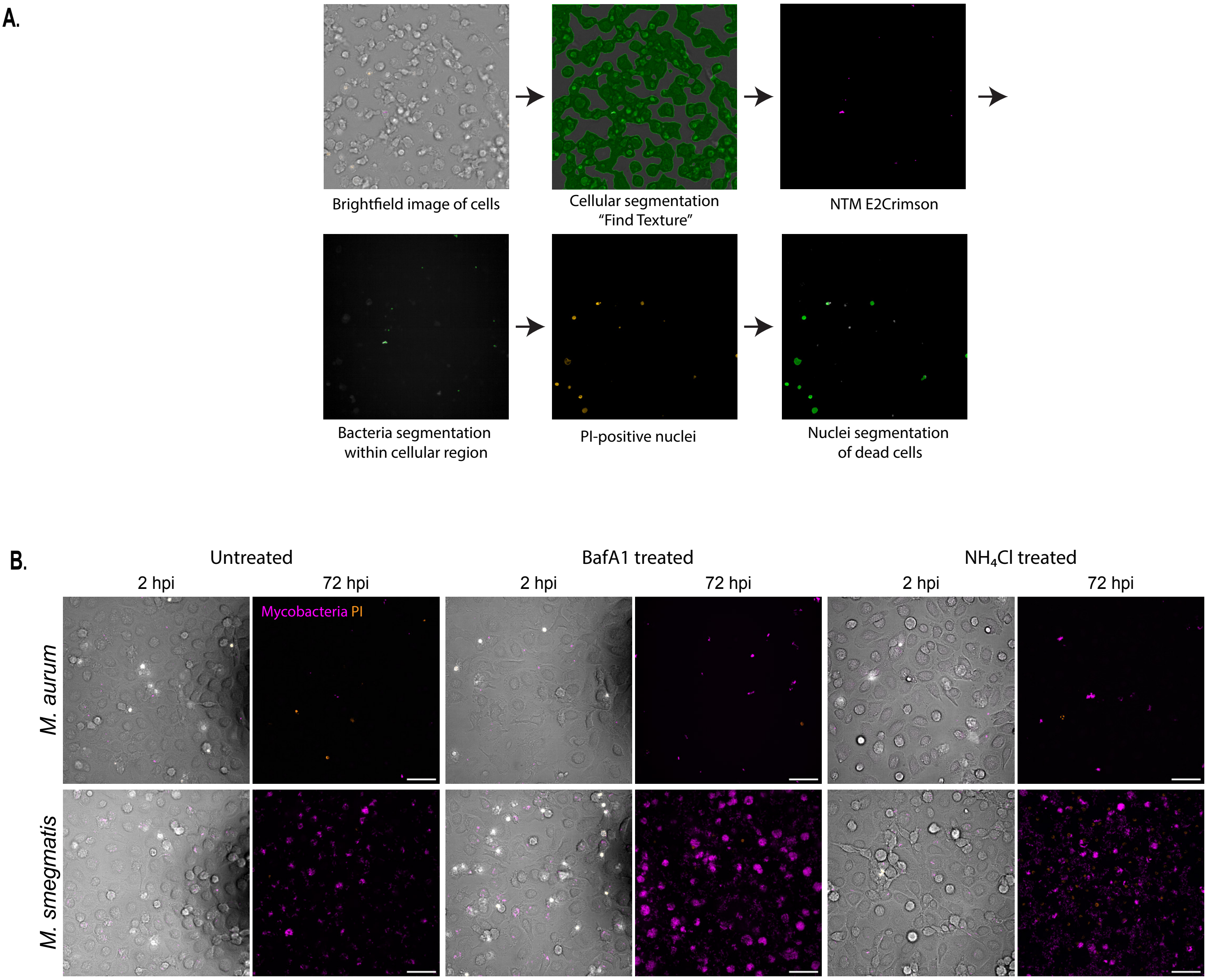

Supplementary Figure 5

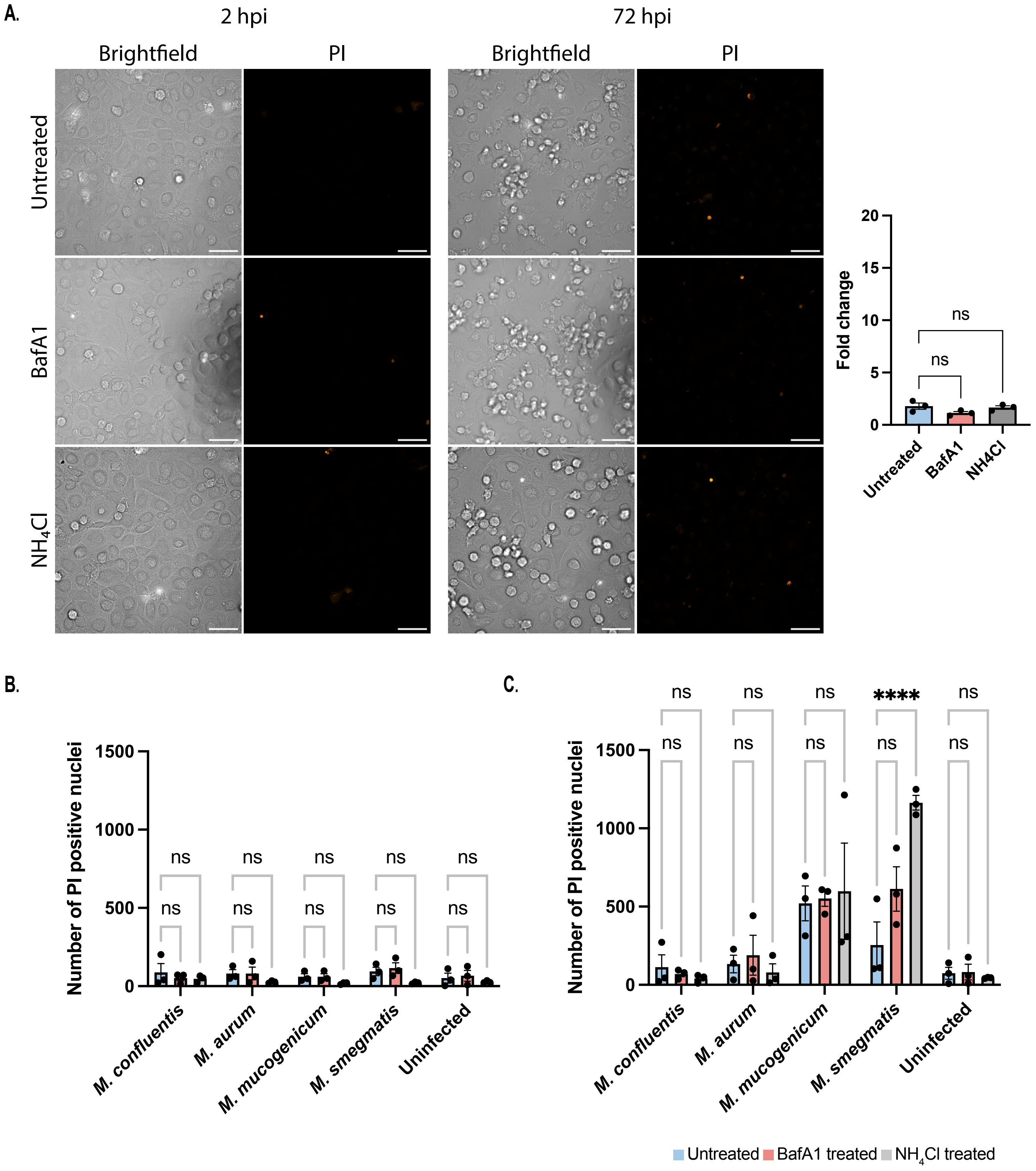

Supplement: Supplementary file 1 — Figure S1: (A) External pH measurements of M. confluentis and M. mucogenicum in 7H9 media in pH conditions 4.5, 5.5, 6.6 and 8.0 with a phosphate citrate buffer. (B) External pH measurements taken during the growth curves of each mycobacterial species in MES and MOPS buffer with the positive control being unbuffered 7H9 media at pH 6.6. Data is representative n = 3 biological replicates ± SEM and visualised on GraphPad Prism v10.1.1. Figure S2: Uptake of mycobacterial species in human monocyte derived macrophages. Representative images showing intracellular fluorescent mycobacterial species (arrows) with extracellular bacteria (arrowheads) detected with an anti‐LAM antibody 2 h post infection. Images were acquired on the Leica SP8 confocal microscope with a maximum z projection ranging from 4.18 to 9.25 μm before analysed in Fiji v1.54f with the orthogonal view of YZ and XZ. Scale bar represents 20 μm. Figure S3: Segmentation workflow of fixed replication imaging experiments. Image acquired is a fixed image of M. mucogenicum 48 h post infection with cell boundaries segmented with galectin 3 Alexa 488. Acquired images were max z‐projections of 4 μm. Infected cells were cells defined as those containing intracellular E2Crimson fluorescent ‘spots’. Segmentation was performed on the Harmony software 4.9. Figure S4: (A) Segmentation workflow of the live cell replication experiment. Image acquired is maximum z projection of a live cell image of M. aurum in untreated HMDMs. (B) Representative images of the live cell replication of M. aurum and M. smegmatis within untreated, NH4Cl and BafA1 treated HMDMs. Images acquired on the OPERA Phenix and segmented on the Harmony software 4.9. Scale bar represents 50 μM. Figure S5: Cell death of untreated, BafA1 treated and NH4Cl treated HMDMs cell over 72 h. (A) Cell death was calculated based on the fold change of the number of propidium iodide (PI)‐positive nuclei between 2 and 72 hpi. The number of PI positive nuclei for each NTM [file MMI-125-375-s002.pdf]
